# Pericardial Manifestations in Systemic Lupus Erythematosus: Clinical Spectrum and Potential Modifying Factors

**DOI:** 10.3390/jcdd13070289

**Published:** 2026-06-23

**Authors:** Mislav Radić, Petra Šimac Prižmić, Tina Bečić, Hana Đogaš, Ivana Jukić, Jonatan Vuković, Damir Fabijanić, Josipa Radić

**Affiliations:** 1 Department of Internal Medicine, Division of Rheumatology, Allergology and Clinical Immunology, Center of Excellence for Systemic Sclerosis in Croatia, University Hospital of Split, 21000 Split, Croatia; psimac@kbsplit.hr; 2 Internal Medicine Department, School of Medicine, University of Split, 21000 Split, Croatia; jonatan.vukovic@mefst.hr (J.V.); josiparadic1973@gmail.com (J.R.); 3 Cardiovascular Disease Department, University Hospital of Split, 21000 Split, Croatia; damirfabijanic62@gmail.com; 4 Department of Neurology, University Hospital of Split, 21000 Split, Croatia; hana.dogas@gmail.com; 5 Internal Medicine Department, Gastroenterology Division, University Hospital of Split, 21000 Split, Croatia; ivjukic@gmail.com; 6 Faculty of Health Sciences, University of Split, 21000 Split, Croatia; 7 Clinical Propaedeutic Department, School of Medicine, University of Split, 21000 Split, Croatia; 8 Department of Internal Medicine, Division of Nephrology, Dialysis and Arterial Hypertension, University Hospital of Split, 21000 Split, Croatia

**Keywords:** systemic lupus erythematosus, pericarditis, pericardial effusion, antiphospholipid syndrome, cardiac involvement

## Abstract

Background: Pericardial involvement is the most common cardiac manifestation of systemic lupus erythematosus (SLE), ranging from mild effusion to recurrent pericarditis and cardiac tamponade. The influence of antiphospholipid syndrome (APS) on lupus-related pericardial disease remains unclear. Methods: A systematic review was conducted in accordance with PRISMA 2020 guidelines and registered in PROSPERO. PubMed, Web of Science, Scopus, and the Cochrane Library were searched from inception to January 2026 for observational studies evaluating pericardial manifestations in adult SLE patients. APS/aPL status was considered a potential modifying factor when reported. Results: Seven observational studies were included. Pericardial involvement ranged from acute and recurrent pericarditis to large effusions and cardiac tamponade. Across studies, it was consistently associated with higher disease activity and markers of immune activation. Recurrent pericarditis emerged as a clinically relevant phenotype linked to more severe disease and worse outcomes. Cardiac tamponade, although rare, was associated with significant morbidity and mortality. APS/aPL-related data were heterogeneous and inconsistently reported across studies. No consistent APS-specific association with pericardial disease could be established, although APS or aPL-related findings were occasionally reported in selected severe or clinically complex presentations. Conclusions: Pericardial involvement in SLE reflects systemic inflammatory burden and spans a broad clinical spectrum. Current evidence regarding APS remains limited and heterogeneous, although APS may contribute to disease complexity in selected severe presentations. Importantly, isolated aPL positivity should not be interpreted as equivalent to formally classified APS. Prospective studies with standardized definitions and systematic assessment of APS are needed.

## 1. Introduction

Systemic lupus erythematosus (SLE) is a chronic, multisystem autoimmune disorder characterized by immune complex deposition, complement activation, and widespread inflammation and vascular damage. Cardiovascular involvement significantly contributes to morbidity and mortality in SLE and encompasses a broad range of features, including accelerated atherosclerosis, myocarditis, valvular disease, pulmonary hypertension, and pericardial involvement [1,2]. Among these, pericardial involvement is the most common cardiac manifestation, occurring at all stages of the disease and ranging from mild, often asymptomatic pericardial effusion to recurrent pericarditis and life-threatening cardiac tamponade [3,4].

Pericarditis in SLE has a variable clinical course that significantly differs from idiopathic or viral pericarditis. While some acute episodes respond to standard anti-inflammatory treatments, many patients experience recurrent or treatment-resistant disease, often linked to high systemic inflammation, immune dysregulation, and other organ involvement [5,6]. Recent population and cohort studies show that lupus pericarditis is more than a benign condition; it is associated with higher recurrence rates and worse long-term outcomes, including reduced survival [4,6]. These findings highlight the importance of improved risk stratification and a better understanding of factors that influence disease progression.

Antiphospholipid antibodies (aPL) and antiphospholipid syndrome (APS) have been implicated in cardiovascular manifestations of SLE; however, their specific relationship with lupus-related pericardial disease remains poorly characterized and inconsistently reported across studies. APS involves thrombo-inflammation, endothelial dysfunction, and microvascular injury, which can enhance pericardial inflammation, sustain effusions, and lead to recurrent or severe clinical presentations [7,8]. Cardiac involvement in APS extends beyond valvular disease and coronary events, encompassing pericardial effusion, pericarditis, and, in rare cases, catastrophic presentations associated with multiorgan thrombosis [9,10]. Despite these mechanistic and clinical links, the modifying role of APS in lupus-related pericardial manifestations has not been systematically synthesized, and existing studies frequently address SLE and APS as separate entities rather than overlapping syndromes.

Advances in cardiac imaging have substantially refined the evaluation of pericardial disease in systemic inflammatory disorders. Transthoracic echocardiography remains the first-line modality for detecting pericardial effusion and assessing hemodynamic compromise; however, it provides limited information on inflammatory activity and tissue characterization [11]. Contemporary multimodal imaging, particularly cardiac magnetic resonance (CMR), enables detailed assessment of pericardial edema, inflammation, fibrosis, and constriction, thereby facilitating differentiation between active, potentially reversible disease and chronic structural remodeling [12]. Recent scientific statements and consensus documents have emphasized the central role of CMR and complementary imaging techniques in managing cardiac involvement in systemic inflammatory diseases, including SLE [13,14]. These advances are particularly important in complex clinical situations, such as recurrent pericarditis, ambiguous symptoms, or suspected microvascular involvement related to APS.

From a therapeutic perspective, management of lupus pericarditis largely relies on evidence from idiopathic pericarditis, with non-steroidal anti-inflammatory drugs, colchicine, and corticosteroids forming the cornerstone of treatment [15,16]. However, autoimmune-mediated pericardial disease often requires individualized immunosuppressive strategies, and emerging data suggest that targeted anti-inflammatory approaches, including interleukin (IL)-1 inhibition, may benefit selected refractory cases [17]. The presence of APS may further complicate management by increasing thrombotic risk and influencing therapeutic decision-making, underscoring the importance of integrated cardiovascular and rheumatologic assessment.

Given clinical heterogeneity, prognostic implications, and the evolving diagnostic and therapeutic landscape, a comprehensive synthesis of pericardial manifestations in SLE is warranted. In particular, there is a need to better understand whether APS and aPL positivity contribute to the presentation, severity, recurrence, and outcomes of lupus-related pericardial disease, and to contextualize these findings within modern multimodal imaging and contemporary management strategies. Therefore, this systematic review aims to summarize current evidence on pericardial involvement in SLE, explore the potential contribution of APS and aPL positivity to disease severity and clinical complexity, and integrate clinical, imaging, and mechanistic insights to inform risk stratification and individualized care.

## 2. Materials and Methods

### 2.1. Study Design and Registration

This systematic review was conducted in accordance with the Preferred Reporting Items for Systematic Reviews and Meta-Analyses (PRISMA) 2020 statement. The review protocol was prospectively registered in the International Prospective Register of Systematic Reviews (PROSPERO; registration ID: CRD420251183100). The review was designed as a systematic review of etiology and risk factors, following the Joanna Briggs Institute methodology for reviews of etiology and risk [18,19].

### 2.2. Literature Search Strategy

A thorough literature search was conducted using PubMed, Scopus, Web of Science, and the Cochrane Library, covering all papers from their inception through January 2026. The search strategy primarily aimed to identify studies on SLE and pericardial involvement, including pericarditis, pericardial effusion, cardiac tamponade, and constrictive pericarditis. APS and aPL were not included as mandatory search terms because the review sought to capture the full spectrum of lupus-related pericardial disease and to assess APS/aPL status as a potential modifying factor rather than as an essential inclusion criterion. This approach was chosen to avoid missing relevant SLE pericardial studies in which APS/aPL data were reported only secondarily or in subgroup analyses. No restrictions were applied regarding publication year. Reference lists of relevant studies and reviews were also manually screened to identify additional eligible articles. The complete database-specific search strategies for all electronic databases are provided in Supplementary Table S1.

### 2.3. Study Selection

**Figure 1:**
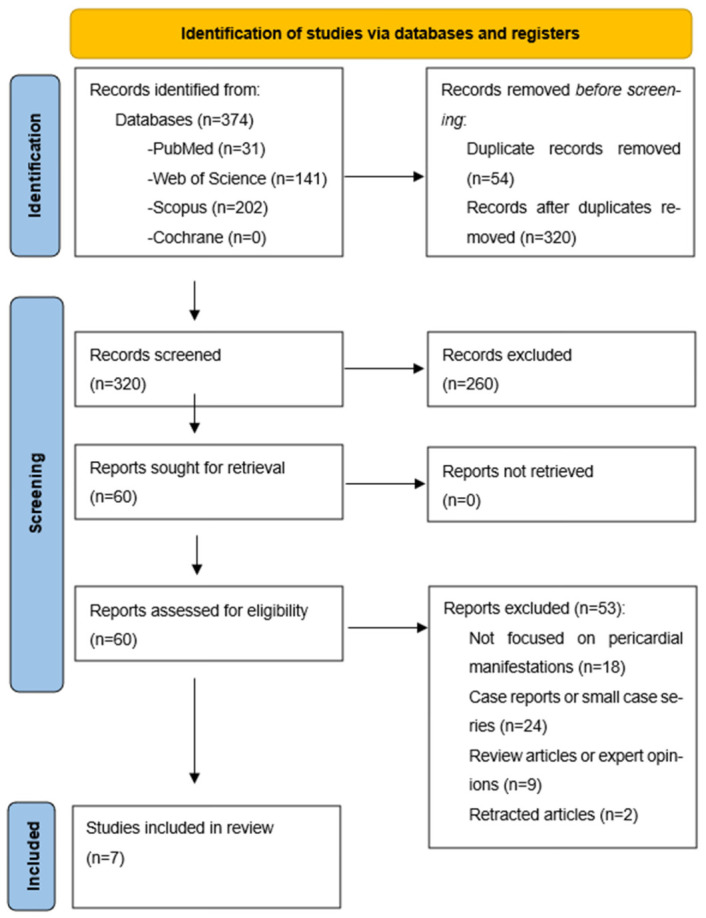
PRISMA 2020 flow diagram of study selection.

Duplicate records were identified and removed before the screening stage. Two reviewers (TB and PŠP) independently screened titles and abstracts to assess eligibility. Full-text articles were then evaluated for inclusion against predefined criteria. Discrepancies between reviewers were resolved through discussion and consensus. The study selection process, including reasons for exclusion at each stage, is summarized in the PRISMA 2020 flow diagram (Figure 1).

### 2.4. Eligibility Criteria

Studies were eligible if they reported original data on adult patients with SLE and assessed pericardial involvement, including pericarditis, pericardial effusion, cardiac tamponade, or constrictive pericarditis. Observational study designs were included, covering cohort, case–control, and cross-sectional studies. Eligible studies needed to report clinical outcomes related to pericardial disease. Studies were excluded if they were case reports or small case series, review articles, editorials, expert opinions, pediatric studies, or non-original research. Small retrospective cohorts focusing on rare but severe pericardial manifestations (e.g., cardiac tamponade) were included if they provided outcome data and met predefined inclusion criteria. Articles that did not focus on pericardial manifestations or lacked sufficient data on outcomes or relevant clinical characteristics were also excluded.

### 2.5. Data Extraction

Data extraction was conducted independently by two reviewers (TB and PŠP) using a standardized extraction form. Extracted data included study characteristics, patient population, type of pericardial manifestation, assessment of APS or aPL, and reported outcomes. APS/aPL status was recorded when available but was neither required for study inclusion nor considered a primary outcome; it was treated as a potential modifying factor. Any discrepancies in data extraction were resolved by consensus. When available, details on the APS definition, aPL profile, or confirmation of aPL persistence were also extracted. The main characteristics of the included studies are summarized in Table 1. Moreover, detailed characterization of APS/aPL assessment, including definitions, antibody profiling, and confirmation of persistence, is presented in Table 2.

### 2.6. Risk of Bias Assessment

**Figure 2:**
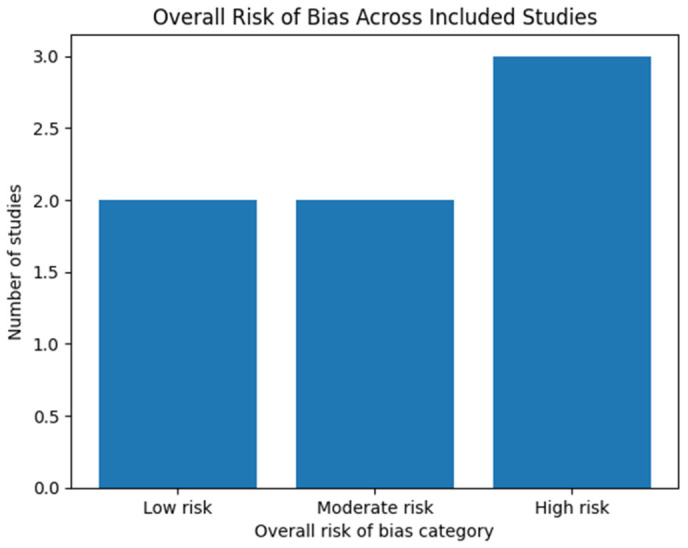
Overall risk of bias across included studies.

Risk of bias was independently evaluated by two reviewers using validated tools appropriate for the study design. The Newcastle–Ottawa Scale was used for cohort and case–control studies, and the Joanna Briggs Institute Critical Appraisal Checklist was used for the cross-sectional study. Overall risk of bias was classified as low, moderate, or high based on selection processes, control of confounding factors, and the assessment of outcomes or exposures. The results of the risk-of-bias assessment are presented in Table 3, with an overall graphical summary in Figure 2. A more detailed domain-level assessment across individual studies is additionally summarized in Table 4.

A formal GRADE-based certainty-of-evidence assessment was not performed because the included studies were exclusively observational, clinically heterogeneous, and not suitable for quantitative synthesis. Instead, methodological quality and risk of bias were assessed using validated study-design–specific appraisal tools.

### 2.7. Data Synthesis

A formal meta-analysis was not performed because statistical pooling was considered methodologically inappropriate. It was also deemed unlikely to yield clinically meaningful summary estimates in the present context. Following re-evaluation of the eligibility criteria, only 7 studies met the final inclusion criteria for a formal qualitative synthesis. The included studies showed substantial clinical and methodological heterogeneity. These differences included variations in study design (prospective cohort, retrospective cohort, case–control, and cross-sectional), patient populations, follow-up duration, and definitions of pericardial involvement. Reported outcomes also differed considerably across studies. They included acute pericarditis, recurrent pericarditis, pericardial effusion, cardiac tamponade, and broader cardiac manifestations. These outcomes were considered clinically non-equivalent constructs for statistical pooling.

In addition, APS and aPL antibody assessment were inconsistently defined and reported. There was variable characterization of antibody profiles, confirmation of persistence, and APS classification criteria. Several studies also lacked standardized effect estimates or comparable quantitative outcome measures required for robust pooled analysis. Collectively, these limitations were likely to reduce the interpretability and reliability of pooled quantitative estimates. Therefore, a structured qualitative narrative synthesis was conducted instead. To improve interpretability, additional quantitative findings, such as frequency measures, recurrence data, survival outcomes, and prognostic associations, were included in the study characteristics tables where available.

In addition, when available, effect estimates reported by individual studies (including hazard ratios, odds ratios, or recurrence-related associations) were qualitatively summarized to improve clinical interpretability despite the absence of formal quantitative pooling.

## 3. Results

### 3.1. Study Selection

The literature search identified 374 records across four electronic databases. After removing 54 duplicates, 320 records were screened by title and abstract, leading to the exclusion of 260. Sixty full-text articles were assessed for eligibility, of which 53 were excluded for predefined reasons. A total of 7 studies met the inclusion criteria and were included in the qualitative synthesis. The study selection process is illustrated in the PRISMA 2020 flow diagram (Figure 1).

### 3.2. Characteristics of Included Studies

The main characteristics of the included studies are summarized in Table 1. The studies were published between 2006 and 2025 and comprised prospective and retrospective cohort studies, a case–control study, and a cross-sectional study. Sample sizes ranged from 28 to approximately 2400 patients with SLE. Studies originated from North America, Europe, and Asia, reflecting diverse patient populations and clinical settings. Pericardial manifestations assessed across studies included acute pericarditis, recurrent pericarditis, pericardial effusion, and cardiac tamponade. Some studies focused specifically on pericarditis [5,20], whereas others addressed severe presentations such as cardiac tamponade [21,22] or evaluated pericardial involvement in broader assessments of cardiac disease in SLE [23].

### 3.3. Spectrum of Pericardial Manifestations in SLE

Acute pericarditis was the most commonly reported pericardial manifestation across the included studies. In a large prospective cohort, Ryu et al. (2017) demonstrated that pericarditis frequently occurs in patients with SLE and is closely linked to higher overall disease activity [5]. Similarly, a case–control study by Hsieh et al. (2023) identified acute pericarditis as a clinically important presentation associated with active immunological disease [20]. Recurrent pericarditis has been identified as a distinct clinical phenotype in larger patient cohorts. Kim et al. (2025), in a comprehensive retrospective cohort study, found that recurrent pericarditis is associated with more severe disease and more frequent inflammatory flares, highlighting its significance within the spectrum of lupus pericardial disease [4].

Severe pericardial manifestations, including large pericardial effusions and cardiac tamponade, were less common but had a substantial clinical impact. Goswami et al. (2018) described cardiac tamponade as a rare but severe complication of SLE, while Rosenbaum et al. (2009) reported high morbidity and mortality among patients presenting with tamponade in a retrospective cohort [21,22].

### 3.4. Clinical and Serological Associations

Several studies have reported associations between pericardial involvement and markers of active lupus. Ryu et al. (2017) [5] found that pericarditis was significantly associated with higher disease activity scores, while Hsieh et al. (2023) [20] demonstrated associations between lupus pericarditis and elevated anti–double-stranded DNA antibody titers and hypocomplementemia. These findings were consistent across study designs and patient populations [5,20]. Regarding outcomes, Chen et al. (2022) [6] reported in a retrospective cohort study that pericarditis in patients with SLE was associated with worse survival, suggesting prognostic implications of pericardial involvement. However, outcome definitions and follow-up durations varied considerably across studies [6]. Clinically relevant prognostic associations and reported effect estimates across the included studies are summarized in Table 5.

### 3.5. Antiphospholipid Syndrome as a Potential Modifying Factor

Assessment of APS or aPL varied across the included studies, as did the definitions of APS. For interpretative purposes, the available evidence was considered narratively in two broad categories: studies evaluating formally classified or clinically recognized APS, and studies reporting isolated or partial aPL positivity without standardized confirmation of APS. Some studies used established classification criteria (e.g., Sapporo or Sydney criteria), whereas others reported only the presence of aPL without meeting clinical APS criteria. APS or aPL status was explicitly reported or analyzed in some studies [4,23], whereas others included these variables only partially or as secondary observations [5,6]. In the cross-sectional study by Amoroso et al. (2006) [23], aPL were linked to cardiac manifestations in SLE; however, pericardial involvement was not evaluated as a separate outcome [23]. Overall, APS was not consistently associated with pericardial disease across studies, but it was occasionally reported in the context of more complex or severe cardiac involvement. Due to heterogeneous reporting and a lack of standardized APS definitions, no definitive conclusions could be drawn regarding its role in lupus pericardial disease.

Overall, the available evidence does not support a consistent or causal association between APS and pericardial manifestations in SLE. The interpretation of APS as a potential disease modifier remains largely hypothesis-generating because APS/aPL assessment was heterogeneous, often incomplete, and frequently based on non-standardized definitions or partial serological data. Consequently, mechanistic interpretations should be viewed cautiously and distinguished from direct empirical evidence.

Across the included studies, the quality of APS characterization varied substantially. Only a minority of studies referenced formal APS classification frameworks, whereas most reported isolated antiphospholipid antibody positivity without systematic confirmation of persistence or fulfillment of clinical criteria. Accordingly, the available evidence can be broadly grouped into two categories: studies evaluating formal or clinically recognized APS and studies reporting isolated or partial aPL positivity. Associations between APS/aPL and pericardial manifestations appeared more suggestive in studies describing clinically complex or severe phenotypes; however, studies limited to isolated aPL positivity often lacked sufficient phenotypic detail to support APS-specific conclusions. This distinction is clinically important because isolated aPL positivity does not necessarily carry the same prognostic or therapeutic implications as definite APS. These findings are summarized in Table 2.

Formal comparison of effect sizes across studies was not possible because most studies reported outcome associations qualitatively or used non-comparable statistical approaches.

### 3.6. Risk of Bias Assessment

The results of the risk-of-bias assessment are shown in Table 2, with an overall graphical summary in Figure 2. Two studies were considered to have a low risk of bias [4,5], two a moderate risk [6,20], and three a high risk [21,22,23]. Studies with a higher risk of bias were mainly small retrospective cohorts focused on rare but severe pericardial conditions, such as cardiac tamponade. These limitations might affect the observed associations and should be taken into account when interpreting the findings. Importantly, studies judged to be at high risk of bias were mainly small retrospective cohorts of rare, severe presentations, and their findings were considered supportive but not definitive. Because APS/aPL-related analyses were frequently secondary and inconsistently characterized, APS-specific interpretations should be considered exploratory rather than definitive.

### 3.7. Summary of Results

Taken together, the included studies demonstrate that pericardial involvement is a frequent and clinically significant manifestation of SLE, spanning a broad clinical spectrum from acute and recurrent pericarditis to life-threatening cardiac tamponade. Across the 7 included studies, acute pericarditis and pericardial effusion were the most commonly reported manifestations, whereas recurrent pericarditis and cardiac tamponade were reported in fewer studies focused on more severe disease phenotypes. Associations between pericardial involvement and higher disease activity, as well as serological markers of immune activation, were consistently reported in larger, methodologically stronger studies. Severe manifestations, although less frequently described, were associated with substantial morbidity and adverse clinical outcomes. In contrast, APS or aPL status was assessed inconsistently and with variable definitions, limiting direct comparisons across studies. Overall, the available evidence supports pericardial disease as a marker of inflammatory burden and disease severity in SLE, while the role of APS remains heterogeneous and context-dependent. Although formal quantitative synthesis was not feasible, several studies reported clinically relevant associations between pericardial manifestations and markers of disease activity, recurrence, or adverse outcomes. Only studies reporting clinically interpretable prognostic or outcome associations were included in the structured summary presented in Table 5.

## 4. Discussion

This systematic review synthesizes available evidence on pericardial manifestations in SLE, with a particular focus on clinical heterogeneity, prognostic implications, and the potential modifying role of APS. Several important observations emerge. First, pericardial involvement is not only the most frequent cardiac manifestation of SLE but also a clinically meaningful disease spectrum, ranging from self-limited acute pericarditis to recurrent, refractory disease and life-threatening cardiac tamponade. Second, pericardial manifestations are consistently associated with systemic disease activity and immunological activation, underscoring their role as markers of inflammatory burden rather than isolated cardiac complications. Third, while APS does not appear to be a uniform predictor of pericardial disease, it may modify disease severity and complexity in selected clinical contexts.

### 4.1. Pericardial Disease as a Marker of Systemic Lupus Activity and Severity

Across the included studies, pericardial involvement was associated with features of active and severe SLE. Large cohort data demonstrated that pericarditis is associated with higher disease activity indices and systemic inflammatory flares [4,5]. Case–control and retrospective studies further supported associations with serological markers of immune activation, including elevated anti–double-stranded DNA antibody titers and hypocomplementemia [6,20]. These findings are consistent with broader reviews of cardiac involvement in SLE, which highlight immune complex deposition and complement-mediated inflammation as key mechanisms underlying serosal involvement [1,2].

Importantly, emerging evidence challenges the traditional perception of lupus pericarditis as a benign or transient manifestation. Population-based data indicate that recurrent pericarditis is not uncommon and is associated with more severe disease phenotypes and repeated inflammatory relapses [4]. Moreover, pericarditis has been linked to adverse survival outcomes in retrospective cohorts, suggesting that it may serve as a surrogate marker of cumulative inflammatory damage or uncontrolled systemic disease [6]. These observations parallel data from autoimmune pericarditis in non-lupus populations, where recurrence and refractoriness are increasingly recognized as markers of poor prognosis [16,24]. Importantly, studies with high risk of bias were predominantly small retrospective cohorts focusing on rare severe manifestations, which may overestimate associations with adverse outcomes. Therefore, conclusions regarding severe pericardial phenotypes should be interpreted cautiously.

### 4.2. Severe Pericardial Manifestations: Effusion and Cardiac Tamponade

Although less common, severe pericardial manifestations, such as large effusions and cardiac tamponade, have substantial clinical implications. Small retrospective cohorts consistently describe tamponade as a rare but life-threatening complication of SLE, often occurring in the setting of high disease activity or delayed diagnosis [21,22,25]. Historical case series and observational reports document notable morbidity and mortality, especially when tamponade is the first sign of SLE or occurs alongside multiorgan involvement [26,27]. Despite their higher risk of bias, the inclusion of these small cohorts in the present review is clinically justified given the rarity and severity of these presentations. Their findings support the idea that lupus-related pericardial disease exists along a spectrum, with severe cases potentially resulting from a combination of an intense immune response, vascular damage, and delayed treatment.

Available pathological and pericardial fluid data on lupus pericarditis remain limited and are largely derived from small observational cohorts and case reports. Reported findings generally reflect inflammatory serositis characterized by immune-mediated inflammation, exudative pericardial effusions, and variable degrees of fibrinous involvement, although systematic histopathological characterization is lacking.

### 4.3. The Modifying Role of Antiphospholipid Syndrome

The role of APS in lupus-related pericardial disease remains complex and incompletely understood. Across the included studies, formal APS classification and isolated aPL positivity were inconsistently evaluated, and standardized definitions were often lacking, limiting the ability to establish a clear, uniform association with pericardial manifestations. Available observations suggest that isolated aPL positivity and APS-related thrombo-inflammatory mechanisms may contribute to selected severe or atypical presentations; however, current evidence remains insufficient to establish a consistent APS-specific association with lupus-related pericardial disease. Rather, APS may represent a potential disease modifier in selected clinical contexts, although this interpretation remains largely inferential and biologically hypothesis-driven. APS is biologically characterized by thrombo-inflammatory pathways, endothelial dysfunction, and microvascular injury, mechanisms that could plausibly contribute to amplification of ongoing pericardial inflammation, persistence of effusions, or more complex disease phenotypes. Nevertheless, these mechanistic considerations extend beyond the direct findings of the included studies and should therefore be interpreted cautiously [7,28]. Observational and cross-sectional studies have reported associations between isolated aPL and cardiac involvement in SLE, including pericardial disease, although these findings are not always specific or isolated [8,23]. In addition, reports of complex clinical presentations involving pericardial effusion, cardiac tamponade, and thrombotic events further support the hypothesis that APS may contribute to more severe or atypical disease expression [9,29].

In the setting of catastrophic APS, cardiac involvement reflects widespread microvascular thrombosis and systemic inflammation, with pericardial disease occurring as part of a multisystem process [10]. Although such presentations are rare, they highlight the potential for APS to influence disease severity and clinical complexity. Taken together, the available evidence suggests that APS does not consistently increase the risk of developing pericardial disease in SLE but may potentially contribute to more severe or clinically complex disease presentations in selected patients. However, current evidence remains insufficient to establish a consistent independent effect of APS on recurrence, severity, or therapeutic refractoriness. The distinction between definite APS and isolated aPL positivity is particularly relevant in clinical interpretation because thrombotic risk, long-term prognosis, and therapeutic considerations differ substantially between these entities.

Consequently, studies reporting only isolated aPL positivity should not be interpreted as equivalent to formally classified APS cohorts.

Importantly, much of the APS-related evidence originated from studies with incomplete phenotyping, inconsistent APS definitions, or isolated reporting of antiphospholipid antibody positivity without confirmation of persistent positivity or fulfillment of formal classification criteria. Therefore, distinctions between definite APS and isolated aPL carrier status were frequently unclear, limiting the strength of conclusions regarding APS-specific effects on pericardial disease.

### 4.4. Role of Imaging in Risk Stratification and Disease Characterization

Although advanced imaging findings were not systematically evaluated in the included studies, evidence from the broader contemporary literature provides important context for risk stratification in lupus-related pericardial disease [13,14,30,31,32,33,34]. Advances in cardiac imaging have reshaped the evaluation of pericardial disease in systemic inflammatory disorders. Transthoracic echocardiography remains essential for initial evaluation, detection of pericardial effusion, and identification of hemodynamic instability, especially in acute settings [11]. However, echocardiography provides limited information on inflammatory activity and tissue characteristics.

CMR imaging has become a pivotal modality for comprehensive pericardial assessment. CMR enables visualization of pericardial edema, inflammation, fibrosis, and constrictive physiology, facilitating differentiation between active, potentially reversible disease and chronic structural remodeling [12]. Typical CMR findings in inflammatory pericardial disease include pericardial thickening, T2-weighted edema, and late gadolinium enhancement reflecting active inflammation. In selected patients, CMR may additionally identify constrictive physiology or concomitant myocardial involvement, which may influence prognosis and therapeutic decision-making. Recent consensus statements and imaging-focused reviews underscore the value of multimodal imaging in guiding therapeutic decisions, particularly in recurrent or refractory pericarditis [13,14]. In SLE and APS, where clinical symptoms may be nonspecific and disease activity fluctuates, advanced imaging may provide critical insights into disease activity and prognosis. Emerging echocardiographic techniques, including global longitudinal strain, further highlight subclinical cardiac involvement that may accompany pericardial disease in SLE [30]. Together, these imaging advances enable a more detailed approach to risk stratification by integrating structural, functional, and inflammatory markers.

### 4.5. Therapeutic Implications and Emerging Strategies

Management of lupus pericarditis largely follows the principles established for idiopathic pericarditis, with non-steroidal anti-inflammatory drugs, colchicine, and corticosteroids forming the therapeutic foundation [15,31]. However, autoimmune-mediated pericardial disease often requires individualized immunosuppressive strategies, particularly in recurrent or steroid-dependent cases [16,32]. Small case series have demonstrated the efficacy of colchicine in treating lupus pericarditis, supporting its role as a cornerstone of therapy [33].

Recent reports suggest that targeted anti-inflammatory therapies, including IL-1 inhibition, may offer benefit in refractory lupus pericarditis, mirroring advances in idiopathic recurrent pericarditis [17]. Recent randomized data from populations with recurrent pericarditis, including contemporary clinical trials evaluating rilonacept, as well as the 2025 ESC Guidelines on pericardial diseases, support IL-1 inhibition as an emerging therapeutic option in selected refractory or corticosteroid-dependent cases [31]. However, evidence specific to SLE-associated or APS-associated pericardial disease remains limited. In patients with concomitant APS, treatment decisions may be further complicated by thrombotic risk, anticoagulation management, and the need to distinguish persistent inflammatory activity from thrombo-inflammatory or microvascular mechanisms. In contrast to idiopathic recurrent pericarditis, lupus-associated pericardial disease frequently occurs in the context of broader systemic immune activation and concomitant organ involvement, which can substantially influence therapeutic decisions. Therefore, the use of IL-1–targeted therapy in SLE requires careful consideration of background immunosuppressive treatment, infection risk, corticosteroid exposure, disease activity in other organ systems, and the potential coexistence of thrombo-inflammatory mechanisms related to APS. In this context, therapeutic decisions should be guided by the severity and recurrence pattern of pericarditis, evidence of active systemic lupus inflammation, concomitant organ involvement, current and cumulative corticosteroid exposure, baseline immunosuppressive burden, infection risk, renal function, pregnancy considerations, and the presence of APS-related thrombotic risk or anticoagulation requirements. In clinical practice, therapeutic escalation often depends not only on the severity or recurrence of pericarditis but also on overall lupus activity, response to conventional immunosuppressive therapy, and the need to balance inflammatory control with long-term treatment toxicity. These factors distinguish lupus-related pericarditis from idiopathic forms and support a more individualized, multidisciplinary management approach. While data remain limited, these emerging approaches highlight the evolving therapeutic landscape and the need for tailored treatment strategies, particularly in patients with coexisting APS, where thrombotic risk must be carefully balanced.

### 4.6. Contextualizing Evidence from the Broader Literature

Beyond the studies included in the present systematic synthesis, a substantial body of literature further contextualizes the clinical relevance and heterogeneity of pericardial involvement in SLE. Narrative reviews, clinical overviews, and guideline documents consistently recognize pericarditis and pericardial effusion as frequent manifestations of inflammatory cardiac disease, while emphasizing differences between autoimmune-mediated and idiopathic forms of pericardial pathology [32,34,35]. General overviews of pericardial effusion further emphasize the multifactorial etiology of pericardial fluid accumulation and highlight the importance of distinguishing inflammatory, autoimmune, infectious, and hemodynamic mechanisms in clinical evaluation and management [36]. Case series and individual reports illustrate that pericardial disease may represent the initial presentation of SLE or occur in atypical clinical contexts, including young patients, pregnancy, or concomitant extracardiac involvement, underscoring the diagnostic challenges and potential for delayed recognition [37,38,39,40]. Additional observational and imaging-based studies have demonstrated that subclinical pericardial and myocardial involvement is common even in asymptomatic SLE patients. Advanced echocardiographic techniques, including strain analysis, may detect early cardiac abnormalities associated with disease activity and cumulative organ damage [30,41,42]. From a systemic perspective, large cohort studies highlight the burden of irreversible organ damage in SLE and reinforce the concept that cardiovascular and serosal involvement contribute to long-term morbidity [43,44]. Within this broader framework, comprehensive reviews of antiphospholipid syndrome further support the biological plausibility of thrombo-inflammatory and microvascular mechanisms influencing cardiac and pericardial manifestations, particularly in complex or multisystem disease presentations [45,46]. In addition to autoimmune and inflammatory etiologies, rare infiltrative disorders may involve the pericardium and mimic systemic autoimmune disease. Erdheim–Chester disease, a rare non-Langerhans cell histiocytosis driven by MAPK pathway mutations, involves the cardiovascular system in approximately 50–70% of patients and may present with pericardial infiltration, pericardial effusion, constrictive physiology, or right atrial pseudotumor [47]. Although ECD is distinct from SLE and APS, awareness of such infiltrative and multisystem disorders is clinically relevant in patients with atypical, treatment-refractory, or diagnostically ambiguous pericardial presentations. Collectively, this complementary evidence aligns with the findings of the present review and reinforces the need to interpret lupus-related pericardial disease within an integrated clinical, immunological, and imaging-based framework.

### 4.7. Limitations and Future Directions

The findings of this review should be interpreted in light of several limitations. The available evidence is heterogeneous, with substantial variation in study designs, sample sizes, outcome definitions, and APS/aPL assessments. Many studies were retrospective, and several that addressed severe pericardial phenotypes had small cohorts and a moderate or high risk of bias. An additional limitation is the inconsistent characterization of APS across studies. In many cohorts, APS definitions were incomplete or not standardized, and antiphospholipid antibody positivity was reported without detailed phenotyping or confirmation of persistence. This limited the ability to distinguish formal APS from isolated aPL positivity and reduced the certainty of APS-specific interpretations. Accordingly, APS-related conclusions in the present review should be interpreted as exploratory and hypothesis-generating rather than confirmatory.

These studies remain clinically informative, particularly for rare manifestations such as cardiac tamponade, but they may overestimate the strength of associations with adverse outcomes and have limited generalizability. APS and antiphospholipid antibody status were also inconsistently reported, limiting the ability to distinguish formal APS from isolated aPL positivity across studies. Future prospective studies should adopt standardized definitions of pericardial disease and APS, systematically confirm aPL persistence, longitudinally assess recurrence and treatment response, and integrate advanced multimodal imaging, particularly CMR. Prospective multicenter cohorts with clearly phenotyped lupus populations would also help distinguish inflammatory from thrombo-inflammatory mechanisms and clarify whether APS independently influences recurrence, severity, or therapeutic refractoriness in lupus-related pericardial disease. Such studies are essential for clarifying the mechanistic links among immune activation, thrombosis, and pericardial inflammation and for informing personalized management strategies.

### 4.8. Clinical Implications

Despite these limitations, the present review highlights several clinically important implications. Pericardial involvement in SLE should be recognized as a marker of systemic disease activity and as a potentially prognostic manifestation. Severe presentations require prompt recognition and appropriate escalation of therapy. From a practical perspective, the available evidence supports a structured clinical approach to SLE patients with pericardial involvement. First, recurrent, severe, or treatment-refractory pericarditis should prompt careful reassessment of overall lupus activity, including serological markers such as double-stranded deoxyribonucleic acid antibodies (anti-dsDNA) and complement consumption [5,6,20]. Second, antiphospholipid antibody testing should be considered, particularly in patients with recurrent pericarditis, persistent or unexplained pericardial effusion, prior thrombotic events, or other features suggestive of APS [8,9,45]. Although formally classified, APS does not appear to be a consistent independent risk factor for pericardial disease; it may increase clinical complexity and influence management decisions in selected patients [7,8,45]. In such cases, treatment strategies may need to integrate anti-inflammatory or immunosuppressive therapy with thrombosis-oriented risk assessment, including consideration of anticoagulation where clinically appropriate [31,32]. In addition, multimodality imaging plays a central role in disease evaluation. Transthoracic echocardiography remains the first-line modality in the acute setting. At the same time, cardiac magnetic resonance imaging is particularly valuable in recurrent, atypical, or diagnostically uncertain cases, allowing differentiation between active inflammatory disease and chronic structural changes [11,12,13,14]. Finally, emerging targeted therapies, in combination with advanced imaging and individualized risk assessment, may offer opportunities to improve outcomes in patients with complex or refractory disease. Overall, while not all patients with lupus-related pericardial disease require APS-directed management, evaluation for APS appears particularly relevant in recurrent, severe, atypical, or treatment-resistant presentations.

## 5. Conclusions

Pericardial involvement is a frequent and clinically relevant manifestation of SLE, reflecting the complex interaction between immune-mediated inflammation, vascular dysfunction, and overall disease activity. Lupus-related pericardial disease is not just a uniformly benign complication; it includes a diverse range of conditions from mild effusions to recurrent pericarditis, and in rare instances, it can be life-threatening tamponade. The available evidence suggests that pericardial manifestations are strongly linked to active and severe SLE and may have significant prognostic implications.

While APS does not consistently increase the risk of pericarditis, available observations suggest that APS/aPL-related thrombo-inflammatory and microvascular mechanisms may contribute to selected severe or clinically complex presentations. These mechanisms provide a biologically plausible framework for considering APS/aPL status in patients with severe or recurrent pericardial disease. However, current evidence remains limited and heterogeneous, and the proposed modifier role of APS should be interpreted cautiously until confirmed in prospective studies with standardized APS assessment.

Advances in cardiac imaging, particularly CMR and multimodality techniques, have significantly enhanced the diagnosis and risk assessment of pericardial disease in SLE. Integration of advanced imaging with clinical and immunological assessment enables more precise differentiation between active inflammatory disease and chronic structural changes, thereby supporting personalized management strategies. Importantly, pericardial involvement should not be regarded as a benign or isolated manifestation, but rather as a clinically meaningful indicator of systemic disease activity and adverse prognosis. While therapeutic approaches continue to rely on conventional anti-inflammatory and immunosuppressive regimens, emerging targeted therapies may offer future options for refractory disease.

Despite the above evidence, current data remain limited by heterogeneity and predominantly by retrospective study designs. Future prospective studies with standardized definitions, systematic assessment of aPL, and incorporation of advanced imaging are needed to clarify mechanisms, refine risk stratification, and optimize treatment. A comprehensive, multidisciplinary approach remains essential for improving outcomes in patients with lupus-related pericardial disease.

## Figures and Tables

**Table 1:** Characteristics of studies included in the systematic review.

Author (Year)	Country	Study Design	Sample Size (SLE)	Pericardial Manifestation	APS/aPL Assessed	Key Outcomes	Main Findings	Quality Notes
Ryu et al. (2017) [5]	USA	Prospective cohort	*n* = 2400	Pericarditis	aPL (partial)	Predictors of pericarditis	Higher disease activity associated with pericarditis	Robust cohort
Kim et al. (2025) [4]	USA	Retrospective cohort	*n* = 1600	Recurrent pericarditis	APS reported	Incidence, recurrence	Recurrent pericarditis associated with severe disease	High-impact, recent
Hsieh et al. (2023) [20]	Taiwan	Case–control	*n* = 80	Acute pericarditis	aPL analyzed	Serological predictors	Anti-dsDNA and hypocomplementemia associated	Moderate size
Chen et al. (2022) [6]	Taiwan	Retrospective cohort	*n* = 73	Pericarditis	APS limited	Survival outcomes	Pericarditis linked to worse prognosis	Single-center
Goswami et al. (2018) [21]	India	Retrospective cohort	*n* = 28	Cardiac tamponade	Not systematic	Clinical course	Tamponade is rare but severe	Small sample
Rosenbaum et al. (2009) [22]	USA	Retrospective study	*n* = 39	Tamponade	APS not primary	Outcomes, treatment	High mortality risk	Older study
Amoroso et al. (2006) [23]	Italy	Cross-sectional	*n* = 82	Cardiac involvement	aPL focus	aPL association	aPL associated with cardiac manifestations	APS-relevant

Abbreviations: SLE, systemic lupus erythematosus; APS, antiphospholipid syndrome; aPL, antiphospholipid antibodies.

**Table 2:** Evaluation of antiphospholipid syndrome and antiphospholipid antibodies in the included studies.

Study	APS/aPL Reported	Definition Used	aPL Panel Reported	Persistence Confirmed	Interpretation for This Review
[5]	Partial aPL data	Not clearly specified	Partial/limited	Not reported	aPL information available, but insufficient for formal APS classification
[4]	APS reported	Not clearly specified	Not fully detailed	Not reported	APS status is considered, but the criteria and laboratory persistence are unclear
[20]	aPL analyzed	aPL-based analysis rather than formal APS classification	Reported variably	Not reported	Supports serological association analysis, not definitive APS classification
[6]	APS limited	Not clearly specified	Limited	Not reported	APS is treated as a contextual variable rather than a robustly phenotyped exposure
[21]	Not systematic	Not reported	Not reported	Not reported	No reliable APS-specific inference is possible
[22]	APS is not the primary focus	Not reported	Not reported	Not reported	APS is not sufficiently characterized
[23]	aPL focus	aPL positivity rather than definite APS	aPL reported	Not reported	Reflects antibody-associated cardiac involvement, not necessarily clinical APS

Abbreviations: APS, antiphospholipid syndrome; aPL, antiphospholipid antibodies.

**Table 3:** Risk of bias assessment of included studies.

Study	Study Design	RoB Tool	Key Bias Domains	Overall Risk of Bias	Main Concerns	Justification
Ryu et al., 2017 [5]	Prospective cohort	NOS	Selection, comparability, outcome	Low	Residual confounding	Large, well-characterized cohort; multivariable adjustment performed
Kim et al., 2025 [4]	Retrospective cohort	NOS	Selection, outcome, follow-up	Low	Retrospective design	High-quality registry data; clear outcome definition; robust statistics
Hsieh et al., 2023 [20]	Case–control	NOS	Selection of controls, exposure ascertainment	Moderate	Control selection; limited adjustment	Appropriate case definition but modest sample size
Chen et al., 2022 [6]	Retrospective cohort	NOS	Outcome assessment, confounding	Moderate	Single-center; limited APS data	Clear outcomes but limited external validity
Goswami et al., 2018 [21]	Retrospective cohort	NOS	Selection bias, small sample	High	Very small cohort; referral bias	Rare outcome study with limited adjustment
Rosenbaum et al., 2009 [22]	Retrospective cohort	NOS	Selection, outcome ascertainment	High	Older design; incomplete confounder control	Historical cohort with limited methodological detail
Amoroso et al., 2006 [23]	Cross-sectional	JBI	Confounding, exposure measurement	Moderate	Temporal ambiguity	APS/aPL assessed, but causality cannot be inferred

Abbreviations: RoB, risk of bias; NOS, Newcastle–Ottawa Scale; JBI, Joanna Briggs Institute; APS, antiphospholipid syndrome; aPL, antiphospholipid antibodies.

**Table 4:** Domain-level risk-of-bias assessment across included studies.

Study	Selection Bias	Exposure Measurement	Outcome Assessment	Confounding	Statistical Analysis	Overall Risk
Ryu et al., 2017 [5]	Low	Low	Low	Moderate	Low	Low
Kim et al., 2025 [4]	Low	Low	Low	Moderate	Low	Low
Hsieh et al., 2023 [20]	Moderate	Low	Low	Moderate	Moderate	Moderate
Chen et al., 2022 [6]	Moderate	Moderate	Low	Moderate	Moderate	Moderate
Goswami et al., 2018 [21]	High	Moderate	Moderate	High	High	High
Rosenbaum et al., 2009 [22]	High	Moderate	Moderate	High	High	High
Amoroso et al., 2006 [23]	Moderate	Moderate	Moderate	Moderate	Moderate	Moderate

**Table 5:** Clinically relevant associations and prognostic findings reported in studies providing evaluable effect estimates or outcome associations.

Study	Outcome Assessed	Reported Association/Effect Estimate	Interpretation
Ryu et al., 2017 [5]	Pericarditis occurrence	African American ethnicity predictive of pericarditis (HR 1.91); anti-Sm and anti-dsDNA associated with pericarditis	Supports the association between inflammatory burden and pericardial disease
Kim et al., 2025 [4]	Recurrent pericarditis	Prednisone is associated with recurrence (RR 1.99); active SLE is associated with recurrence (RR 1.55)	Suggests prognostic relevance of recurrence and disease activity
Hsieh et al., 2023 [20]	Acute pericarditis	Lymphocytopenia associated with pericarditis (OR 2.015); aPL positivity associated with pericarditis (OR 1.569)	Supports a relationship with active immunological disease
Chen et al., 2022 [6]	Survival outcomes	Pericarditis associated with increased mortality (HR 1.963; 95% CI 1.315–2.963)	Indicates prognostic significance of lupus pericarditis
Amoroso et al., 2006 [23]	Cardiac involvement and aPL	aPL positivity associated with cardiac abnormalities (OR 6.1)	Suggests possible APS/aPL-related disease complexity
Goswami et al., 2018 [21]	Cardiac tamponade	Large effusion (>20 mm) predictive of tamponade (OR 93.2); mortality 8.3%	Supports the association between severe effusion and tamponade risk
Rosenbaum et al., 2009 [22]	Cardiac tamponade outcomes	Tamponade occurred in 21.9%; low C4 associated with tamponade (*p* = 0.05); pericardial window required in 55.5%	Supports high morbidity and severe clinical course of lupus-associated tamponade

Abbreviations: APS, antiphospholipid syndrome; aPL, antiphospholipid antibodies; SLE, systemic lupus erythematosus; HR, hazard ratio; OR, odds ratio; RR, risk ratio.

## Data Availability

Data are available on request to the corresponding authors.

## Supplementary Materials

The following supporting information can be downloaded at: https://www.mdpi.com/article/10.3390/jcdd13070289/s1, Supplementary Table S1 contains the complete database search strategies.

## Abbreviations

The following abbreviations are used in this manuscript:

APS	Antiphospholipid syndrome
aPL	Antiphospholipid antibodies
CMR	Cardiac magnetic resonance
CRP	C-reactive protein
dsDNA	Double-stranded deoxyribonucleic acid
IL	Interleukin
JBI	Joanna Briggs Institute
PRISMA	Preferred Reporting Items for Systematic Reviews and Meta-Analyses
RoB	Risk of bias
SLE	Systemic lupus erythematosus
