# Supplementary material for: Pericardial Manifestations in Systemic Lupus Erythematosus: Clinical Spectrum and Potential Modifying Factors
Source: J Cardiovasc Dev Dis. 2026 Jun 23;13(7):289. doi: 10.3390/jcdd13070289 (PMC13409825; doi:10.3390/jcdd13070289)
Supplement: Supplementary file 1 [file jcdd-13-00289-s001.zip › Supplementary Table S1.pdf]

Supplementary Table S1. Complete Database Search Strategies

The following database-specific search strategies were used for the systematic review of pericardial manifestations in systemic lupus erythematosus (SLE). Searches were conducted from database inception through January 2026. No publication year restrictions were applied. Only English-language articles involving human adult populations were considered eligible during the screening process. Reference lists of relevant studies and reviews were also manually screened to identify additional eligible articles.

PubMed Search Strategy

Final search performed: January 2026

Query #1

("Systemic Lupus Erythematosus"[MeSH]

OR "systemic lupus erythematosus"[Title/Abstract]

OR SLE[Title/Abstract])

Query #2

("Antiphospholipid Syndrome"[MeSH]

OR "antiphospholipid syndrome"[Title/Abstract])

Query #3

("Pericarditis"[MeSH]

OR pericarditis[Title/Abstract]

OR "pericardial effusion"[Title/Abstract]

OR "cardiac tamponade"[Title/Abstract]

OR "constrictive pericarditis"[Title/Abstract])

Final Search Strategy

(#1 AND #3) OR (#2 AND #3)

Filters applied: English language; Human studies; Adult population

Scopus Advanced Search Strategy

Final search performed: January 2026

```
(
 ( TITLE-ABS-KEY ( "systemic lupus erythematosus" )
 OR TITLE-ABS-KEY ( SLE ) )
 AND
 ( TITLE-ABS-KEY ( pericarditis )
 OR TITLE-ABS-KEY ( "pericardial effusion" )
 OR TITLE-ABS-KEY ( "cardiac tamponade" )
 OR TITLE-ABS-KEY ( "constrictive pericarditis" ) )
)
OR
(
 TITLE-ABS-KEY ( "antiphospholipid syndrome" )
 AND
 ( TITLE-ABS-KEY ( pericarditis )
 OR TITLE-ABS-KEY ( "pericardial effusion" )
 OR TITLE-ABS-KEY ( "cardiac tamponade" )
 OR TITLE-ABS-KEY ( "constrictive pericarditis" ) )
)
```

Filters applied: English language; Human studies; Adult population.

Web of Science Search Strategy

Final search performed: January 2026

Query #1

((TI=("systemic lupus erythematosus") OR TI=(SLE))

OR (AB=("systemic lupus erythematosus") OR AB=(SLE)))

Query #2

(TI= ("antiphospholipid syndrome")

OR AB = ("antiphospholipid syndrome"))

Query #3

((TI=(pericarditis)

OR TI= ("pericardial effusion")

OR TI= ("cardiac tamponade")

OR TI= ("constrictive pericarditis"))

OR

(AB=(pericarditis)

OR AB= ("pericardial effusion")

OR AB= ("cardiac tamponade")

OR AB= ("constrictive pericarditis")))

Final Search Strategy

(#1 AND #3) OR (#2 AND #3)

Filters applied: English language; Human studies

Cochrane Library Search Strategy

Final search performed: January 2026

(

("systemic lupus erythematosus" OR SLE)

OR

("antiphospholipid syndrome")

)

AND

(

pericarditis

OR "pericardial effusion"

OR "cardiac tamponade"

OR "constrictive pericarditis"

)

Filters applied: Human studies; English language.
